# Coherent Activity in Bilateral Parieto-Occipital Cortices during P300-BCI Operation

**DOI:** 10.3389/fneur.2014.00074

**Published:** 2014-05-15

**Authors:** Kouji Takano, Hiroki Ora, Kensuke Sekihara, Sunao Iwaki, Kenji Kansaku

**Affiliations:** ^1^Systems Neuroscience Section, Department of Rehabilitation for Brain Functions, Research Institute of National Rehabilitation Center for Persons with Disabilities, Tokorozawa, Japan; ^2^Department of Systems Design and Engineering, Tokyo Metropolitan University, Tokyo, Japan; ^3^Cognition and Action Research Group, Human Technology Research Institute, National Institute of Advanced Industrial Science and Technology (AIST), Tsukuba, Japan; ^4^Brain Science Inspired Life Support Research Center, The University of Electro-Communications, Tokyo, Japan

**Keywords:** BMI, magnetoencephalography, imaginary coherence, P300 speller, chromatic stimuli

## Abstract

The visual P300 brain–computer interface (BCI), a popular system for electroencephalography (EEG)-based BCI, uses the P300 event-related potential to select an icon arranged in a flicker matrix. In earlier studies, we used green/blue (GB) luminance and chromatic changes in the P300-BCI system and reported that this luminance and chromatic flicker matrix was associated with better performance and greater subject comfort compared with the conventional white/gray (WG) luminance flicker matrix. To highlight areas involved in improved P300-BCI performance, we used simultaneous EEG–fMRI recordings and showed enhanced activities in bilateral and right lateralized parieto-occipital areas. Here, to capture coherent activities of the areas during P300-BCI, we collected whole-head 306-channel magnetoencephalography data. When comparing functional connectivity between the right and left parieto-occipital channels, significantly greater functional connectivity in the alpha band was observed under the GB flicker matrix condition than under the WG flicker matrix condition. Current sources were estimated with a narrow-band adaptive spatial filter, and mean imaginary coherence was computed in the alpha band. Significantly greater coherence was observed in the right posterior parietal cortex under the GB than under the WG condition. Re-analysis of previous EEG-based P300-BCI data showed significant correlations between the power of the coherence of the bilateral parieto-occipital cortices and their performance accuracy. These results suggest that coherent activity in the bilateral parieto-occipital cortices plays a significant role in effectively driving the P300-BCI.

## Introduction

The brain–machine interface (BMI) or brain–computer interface (BCI) is an interface technology that uses neurophysiological signals from the brain to control external machines or computers (1–3). Electroencephalography (EEG), in which neurophysiological signals are recorded using electrodes placed on the scalp, represents the primary non-invasive methodology for studying BMI.

Our group has used EEG and developed a BMI-based system for environmental control and communication (4). In our system, we modified a P300 speller (5). The P300 speller uses the oddball paradigm and involves the presentation of a selection of icons arranged in a matrix. According to this protocol, the participant focuses on one icon in the matrix as the target, and each row/column or individual icon in the matrix is then intensified in a random sequence. The targets are presented as rare stimuli (i.e., the oddball paradigm). We elicited P300 responses to the target stimuli and then extracted and classified these responses with respect to the target.

We prepared a green/blue (GB) flicker matrix because this color combination was considered the safest in a photosensitive epilepsy study (6). We showed that the GB flicker matrix was associated with a better subjective feeling of comfort than was the white/gray (WG) flicker matrix, and we also found that the GB flicker matrix was associated with better performance (7, 8). The BMI system was used satisfactorily by individuals with cervical spinal cord injury (9, 10).

To highlight areas involved in improving P300-BCI performance, we used simultaneous EEG–fMRI recordings; that is, we sought to identify brain areas that showed greater enhancement in the GB flicker matrix than in the WG flicker matrix. The P300 in the EEG data was detected under both conditions, and the peak amplitudes were larger at the parietal and occipital electrodes, particularly in the late components, under the GB condition than under the WG condition. fMRI data showed activation in the bilateral parietal and occipital cortices, and these areas, particularly those in the right hemisphere, were more activated under the GB condition than under the WG condition. The parietal and occipital regions more involved under the GB condition were among the areas involved in conventional P300s, suggesting the importance of the parietal and occipital regions, especially in the right hemisphere, for the operation of P300-BCI with the GB flicker matrix (11).

Our fMRI–EEG study suggested the importance of the parietal and occipital regions, especially in the right hemisphere, for the operation of P300-BCI with the GB flicker matrix. Analysis of coherence between these regions was expected to show how these regions cooperate in enhancing task performance (12). However, we did not conduct a detailed investigation of coherent activity in these areas because the EEG data were recorded from 27 channels, and the data included severe artifacts due to fMRI scanning. Thus, it was difficult to investigate coherent regional activity in detail.

In this study, we used 306-channel whole-head magnetoencephalography (MEG), which has high spatial resolution compared with EEG, to investigate coherent activity in these areas during the P300-BCI operation. In the coherence analysis, we specifically focused on the alpha band because it has been suggested to be involved in attentional mechanisms (13, 14). In fact, alpha-band oscillation is relevant to visual attention (15–17). Treder and colleagues showed that the alpha power of EEG signals (Po3, Po4) during eye closing in a session before BCI operation was positively correlated with the accuracy of the BCI operation (18). Furthermore, a meaningful shape induces greater alpha-band coherence than a meaningless shape (19).

To capture the coherent activities, we first applied sensor-based analysis. The sensor position and angle can be rearranged for a virtual sensor. We further used a narrow-band adaptive spatial filter to transform the sensor to voxels. We used the imaginary coherence of MEG signals between voxels to investigate functional connectivity when the P300 speller was used. Imaginary coherence uses the imaginary part of the coherence between the channels or between the voxels. It can remove spurious results due to leakage of the imaging algorithm, and thus gives more accurate results without blur (20–22). In recent clinical studies, imaginary coherence was utilized in the preoperative MEG evaluation (23, 24), and these studies have suggested the biological significance of imaginary coherence for evaluating functional connectivity. To highlight brain area(s) that may help improve P300-BCI accuracy, we used mean imaginary coherence (MIC) (25). In our previous EEG–fMRI study, we showed that greater activity was elicited in the right inferior parietal lobule by GB than by WG flicker (11). Based on that study, here, we defined a spherical region of interest (ROI) at the coordinates in the parietal area.

Finally, we reanalyzed some previous EEG-based P300-BCI data, and further investigated the coherence of the power spectrum between bilateral parieto-occipital cortices and performance accuracy.

## Materials and Methods

### Subjects

Thirteen healthy subjects (mean age: 22.9 years, all men, right-handed) participated. One subject’s data were rejected because of excessive noise. All subjects were neurologically normal and right-handed according to the Edinburgh Inventory.

This study received approval from the Institutional Review Board at the National Rehabilitation Center for Persons with Disabilities, Tokorozawa, Japan. All subjects provided written informed consent according to institutional guidelines.

### Task

During the experiment, each participant sat on a chair. Visual stimuli were projected on a screen located in front of participants. The stimulus and triggers that indicated the onset of each trial were presented using Presentation (Neurobehavioral Systems, Inc., Albany, CA, USA).

**Figure 1:**
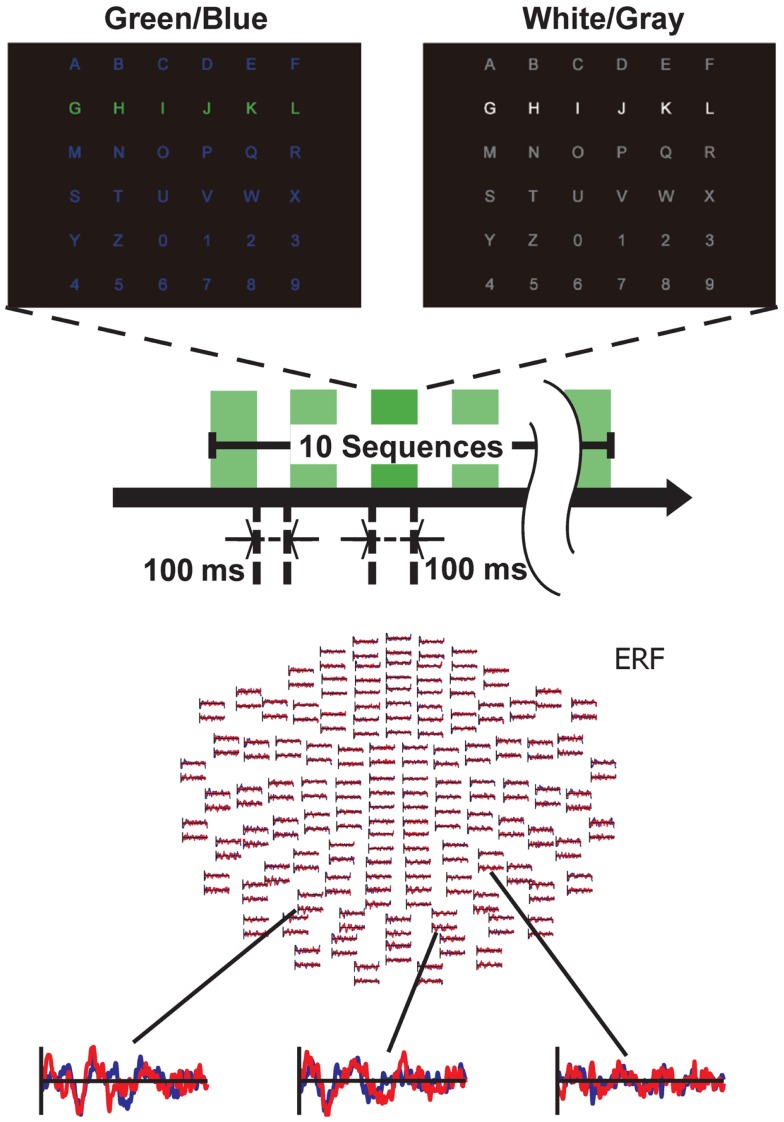
**Experimental setting**. Two types of matrices were used: one for the green/blue (GB) condition and the other for the white/gray (WG) condition. Stimuli were presented in chromatic (GB) and luminance (WG) flickering patterns. Subjects were required to gaze at and attend to the target letter. Event-related fields from a representative subject under the GB condition are shown. The red lines indicate the averaged ERF elicited by the non-target stimuli, whereas the blue lines show the averaged ERF elicited by the target stimuli.

We used two types (GB luminance chromatic condition and WG luminance condition) of visual-flicker stimuli in a 6 × 6 alphabet flicker matrix, modified from the “P300 speller” (5). We prepared a white (20 cd/cm)/gray (6.5 cd/cm) matrix for the WG condition, and a green (20 cd/cm)/blue (6.5 cd/cm) matrix for the GB condition; the luminance was measured using a chromatic meter (CS-200, Konica Minolta Sensing, Inc., Osaka, Japan) on the computer screen, as in our previous study (11). That is, the same luminance change was used under both GB and WG conditions as in the study. We used 100 ms of intensification and 100 ms of rest, because recent visual P300-BCI studies have usually used about 125–300 ms for stimulus-onset asynchrony (SOA) to facilitate rapid communication. Each row/column of the matrix was intensified in random order (Figure 1).

One complete cycle of six rows and six columns constituted a sequence (two target stimuli and 10 non-target stimuli), and 10 sequences constituted a trial. During a trial, participants were asked to focus on one of the icons as the target in the matrix. The target stimuli were presented as rare stimuli (20 target stimuli and 100 non-target stimuli in one trial) to elicit P300 responses (i.e., the oddball paradigm). We conducted six trials during a session under both the GB and WG conditions and simultaneously recorded MEG signals during each session. The order of the experimental conditions (GB or WG) was counterbalanced among participants. We thus recorded 120 segmented data points of the target and 600 segmented data points of the non-target from each participant in each condition.

### MEG data acquisition

For 306-channel whole-head MEG recording, we used a Neuromag Vectorview (Elekta AB, Helsinki, Finland). This system has 102 sensor triplets, with each triplet containing one magnetometer and two gradiometers. Brain activities were sensed and digitized at a rate of 1000 Hz and filtered with a band-pass filter of 0.1–330 Hz. Additionally, four head position indicator (HPI) coils were placed on the subjects’ scalp to record head position relative to the MEG helmet at the beginning of each session. Three cardinal points (nasion, left and right preauricular) were digitized and used for co-registration with structural MRI data and spatial filtering.

### MEG data analyses

#### Sensor-based analyses

We preprocessed the wave data from 204 gradiometers, extracted using FieldTrip (Donders Centre for Cognitive Neuroimaging). These data were then filtered with Signal Space Projections (SSP) and 50-Hz notch and 100-Hz low-pass filters. As described in our previous studies (8, 10), filtered data from 800 ms of the MEG were segmented, starting at 100 ms before intensification. Data from the initial 100 ms (just before the intensification) were used for baseline correction.

**Figure 2:**
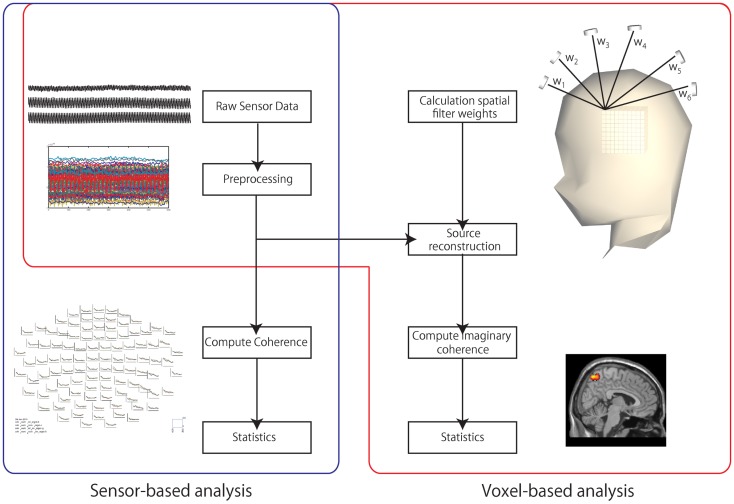
**Data processing**. Two processes were used: sensor-based analysis and voxel-based analysis. In the sensor-based analysis, the coherence between sensors was computed. In the voxel-based analysis, the sensor data were first projected to voxel space. Second, the mean imaginary coherence was computed. Finally, the projected data were transformed to a standard brain.

We then computed the coherence using FieldTrip software (26). The coherence between left and right parieto-occipital channels was computed from the segmented data using only 204 gradiometers. The alpha band (8–12 Hz) was used as the target frequency. We computed the power spectrum density and cross-spectrum density. We combined the 204 gradiometers into 102 channels by calculating the norm. Using these spectra, we computed coherence between channels limited to the bilateral parieto-occipital area. Thus, the number of the channel combinations was 196 (14 × 14 channels). Then, we tested the differences in the coherence between the GB and WG conditions using a paired-sample *t*-test with a Bonferroni correction (Figure 2).

#### Voxel-based analyses

We preprocessed the wave data from the 204 gradiometers and 102 magnetometers using FieldTrip software. These data were filtered with SSP and 50-Hz notch and 100-Hz low-pass filters. As described in our previous studies (8, 10), filtered data from 800 ms of the MEG were segmented, starting at 100 ms before intensification. Data from the initial 100 ms were used for baseline correction. We specifically focused on around 10 Hz for analysis as explained in the Section “Introduction.”

To project the sensor data to voxel space, we used an adaptive beamformer (27) to transform to individual voxel space. The T1 MRI of each participant was used for co-registration. The voxel size was 7.7 mm × 7.7 mm × 7.7 mm. To clarify functional connectivities, we applied imaginary coherence (20–22). Imaginary coherence allows unambiguous detection of brain interaction from EEG/MEG data by retaining only the imaginary part of the cross-spectrum. In the voxel-based analysis, we evaluated the MIC as an index of functional connectivity. MIC is the average coherence between a seed voxel and all the other voxels across the entire brain (≥0). With MIC, we estimated the magnitude of connectivities between the seed voxel and all the other voxels. Voxel data were normalized using SPM8 in MATLAB (MathWorks, Natick, MA, USA). We used the canonical MRI data to create a transformation matrix. We computed MIC from the segmented data of target and non-target for all voxels under each condition. We mapped the difference between MIC of the target and non-target at all voxels (MIC map). We evaluate the differences in the MIC map between the GB and WG conditions. In our previous EEG–fMRI study, we showed greater activation in the parieto-occipital areas under chromatic conditions, and we observed the peak fMRI activation at (*x* = 32, *y* = −53, *z* = 41) (11). We thus defined a 10-mm-radius spherical ROI following the study. Differences between the two MIC maps (GB versus WG) obtained from each individual (*n* = 12) were tested using a paired-sample *t*-test (Figure 2).

### Re-analyses of P300-BCI EEG data

We re-analyzed previous P300-BCI EEG data (*n* = 20) to evaluate relationships between coherence and accuracy with the P300-BCI. The data used in this reanalysis have been published previously (8, 10). In these studies, we prepared an 8 × 10 hiragana (a Japanese character) matrix for the P300 speller, and subjects were required to input 15 hiragana characters with P300-BCI under GB and WG conditions. These data were recorded from eight electrodes (Fz, Cz, P3, Pz, P4, PO7, Oz, and PO8). The data were segmented in the same manner as in the MEG experiments, and the same target frequency was used. The segmented data were also used for P300-BCI evaluation. We used the waveform as a feature vector. The segmented data using a sampling rate of 21 Hz correspond to 15 data points, and data were collected with eight EEG channels. Thus, the feature vector had 120 dimensions. We used Fischer’s linear discriminant analysis for the feature vector to obtain classification accuracy. We evaluated the correlation between the coherence of the EEG signals and accuracy of the P300-BCI.

## Results

### MEG data analyses

#### Sensor-based analyses

**Figure 3:**
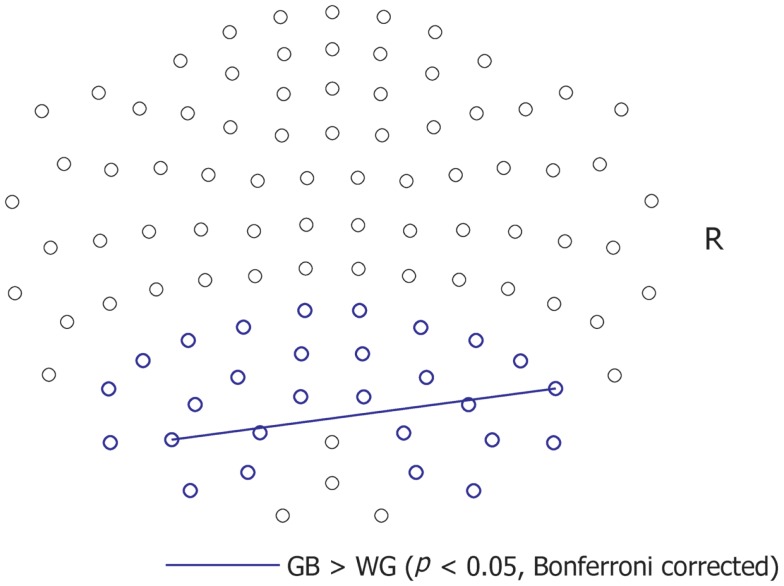
**Sensor-based coherence**. The blue lines indicate significantly greater coherence between sensors under the GB than under the WG condition. Significantly greater coherence (*p* < 0.05, with Bonferroni correction) under the WG than under the GB condition was not observed.

We analyzed the alpha-band coherence between MEG sensors of the bilateral parieto-occipital areas (Figure 3). We set the ROI in the parieto-occipital area because P300 responses had been preferentially observed in these areas according to previous reports using EEG and fMRI data (11, 28–31). Further, there are reports showing alpha-band parieto-occipital activation and coherence in response to meaningful visual stimuli (19).

The alpha-band coherence between a sensor combination [ch2622 + 2623 and ch1732 + 1733, two-tailed *t*(11) = 8.07, *p* = 0.0006, Bonferroni-corrected] was significantly greater under the GB condition than under the WG condition. In contrast, significantly greater coherence was not observed under the WG condition compared to the GB condition.

#### Voxel-based analyses

**Figure 4:**
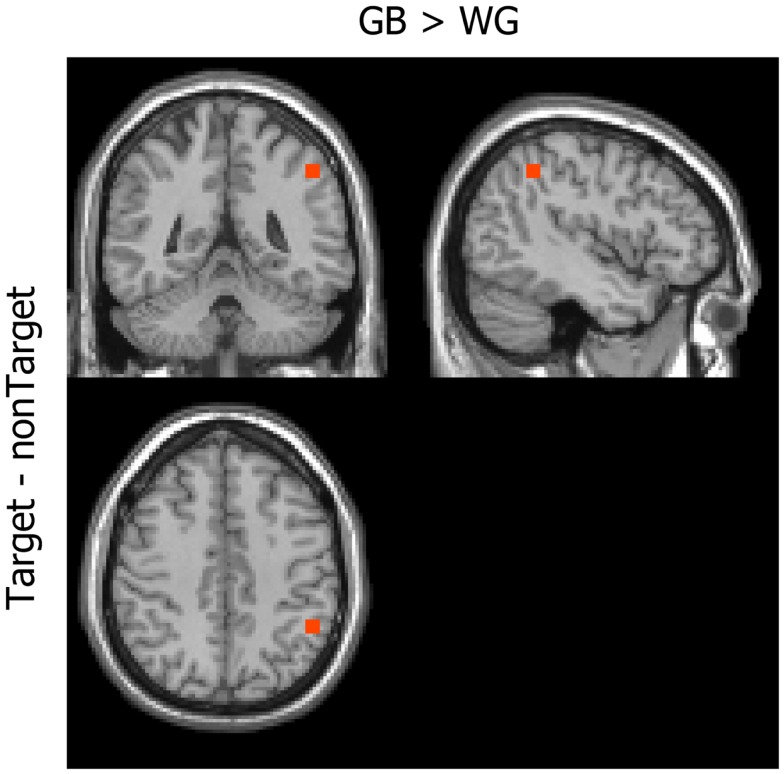
**Mean imaginary coherence**. The inferior parietal area showed higher MIC under the GB than under the WG condition (GB > WG).

Figure 4 shows the difference in the MIC of the power between the GB and WG conditions. Significantly greater coherence was observed in the right inferior parietal lobule under the GB [*x* = 42, *y* = −48, *z* = 44; two-tailed *t*(11) = 3.60, *p* = 0.0253 Bonferroni-corrected] than under the WG condition. The power distribution in this voxel did not show significant difference [two-tailed *t*(11) = 0.05, *p* = 0.9595, uncorrected]. In the WG condition, no coherence greater than that of the GB condition was observed [two-tailed *t*(11) = 0.01, *p* = 1.0, uncorrected].

### Re-analyses of P300-BCI EEG data

**Figure 5:**
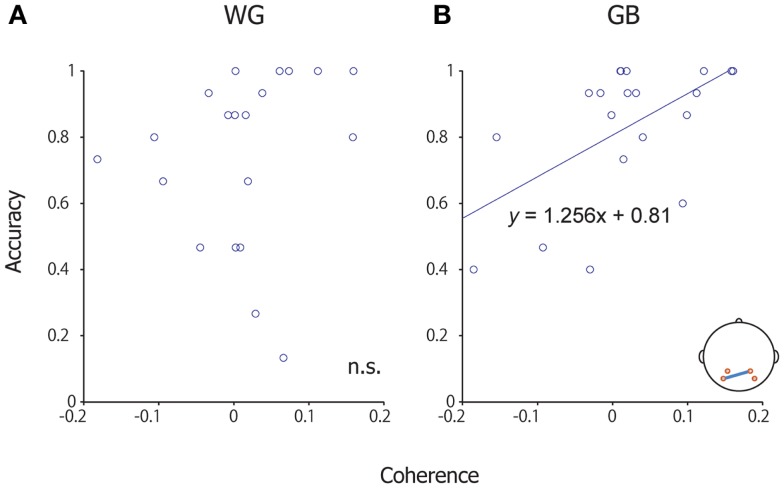
**Scatter plot of coherence and accuracy**. **(A)** Scatter plot of coherence and accuracy under the WG condition. **(B)** Scatter plot of coherence and accuracy under the GB condition. The horizontal axis shows coherence between P4 and Po7, and the vertical axis shows accuracy during the P300-BCI task. A significant correlation was observed under the GB condition.

We re-analyzed the P300-BCI EEG data (8, 10) and computed the coherence between left (P3, PO7) and right channels (P4, PO8); this was done because, in our previous studies, we observed that the electrodes in the parieto-occipital areas play an important role in the P300-BCI operation. We performed a coherence analysis with the EEG data too because a close relationship between MEG data coherence and EEG data coherence has been reported (32). The change in coherence from the non-target to the target condition was used for analysis. A significant correlation was observed between the mean accuracy of each subject and the difference in coherence (target–non-target) (between P4 and PO7; Pearson’s coefficient of correlation, *p* = 0.009, uncorrected) under the GB condition (Figure 5). No significant correlation was observed between other channel combinations (P3 and P4, P3 and PO8, PO7 and PO8). No significant correlation was observed during WG conditions.

We further investigated the relationship between coherence and correct/incorrect responses at the single-trial level. We calculated the coherence between P4 and PO7 in each trial, and then the data were evaluated separately with respect to correct or incorrect responses. This analysis revealed a significant difference between the correct and incorrect responses in the GB condition [two-tailed *t*(298) = 3.80, *p* < 0.001], but not in the WG condition [two-tailed *t*(298) = 1.79, *p* = 0.0746].

## Discussion

Magnetoencephalography activity during P300-BCI use was investigated. When comparing functional connectivity between the right and left occipital channels, significantly greater functional connectivity in the alpha band was observed under the GB than under the WG flicker matrix condition. Application of MIC revealed that coherence was significantly greater in the right posterior parietal cortex under the GB than under the WG condition. Re-analysis of previous EEG-based P300-BCI data showed a significant correlation between the power of the coherence between bilateral parieto-occipital electrodes and performance accuracy.

### Coherent activity and GB flicker

In the sensor-based analysis, by specifically focusing on the alpha band, according to former reports (13, 14, 19), greater coherence between bilateral parieto-occipital areas was observed under the GB than under the WG condition. This result suggests that chromatic stimuli elicited activity in the parietal attentional system more effectively than conventional WG stimuli did. These results are also consistent with our previous study, in which we showed that EEG channels in the lateral parietal areas were more important for P300-BCI operation (33).

We also investigated differences between the GB and WG conditions in the MIC of the power. Significantly greater coherence was observed in the right parieto-occipital area under the GB than under the WG condition (11). In a previous fMRI study, chromatic visual stimuli activated the right occipital and parietal areas (34). In our earlier study, we made simultaneous EEG–fMRI recordings during GB and WG conditions, and the peak of the positive wave in the EEG data was detected under both conditions. The peak amplitudes were larger at the parietal and occipital electrodes, particularly in the late components, under the GB versus the WG condition. fMRI data showed activation in the bilateral parietal and occipital cortices, and these areas, particularly in the right hemisphere, were more activated under the GB than under the WG condition (11). As discussed in that paper, the right hemisphere may be more involved in color detection. Indeed, a psychophysical study suggested the superiority of the right hemisphere for detecting color (35).

### P300-BCI accuracy and coherent activity

Increasing accuracy in P300-BCI operation is a major topic in this field of research. The classification methods of EEG and the optimization of the target of classification have been investigated extensively, and analyses relying on stepwise LDA (36), ICA (37) and common spatial patterns have been applied. Other studies have tested various aspects of visual stimuli, such as their position, pattern, and number to increase accuracy (38–41).

Our re-analysis of previous EEG-based P300-BCI data showed a significant correlation between the power of the coherence between the bilateral parieto-occipital cortices and performance accuracy, suggesting that increasing the power of the coherence between bilateral parieto-occipital cortices may be key to increasing the accuracy of the P300-BCI operation. GB flicker may contribute to increasing the power of the coherence. In this study, we investigated the effects of chromatic stimuli for the P300-BCI, and we found coherence in the right inferior parietal lobule. These coherence features may be useful for classification to improve the accuracy of the P300-BCI, and the coherence values may be usable for neurofeedback training with the P300-BCI.

There are several limitations to this study. First, we were not able to evaluate P300-BCI accuracy during the MEG experiments because, as reported in a previous MEG–BMI study (42), the sensor location showing the highest P300-BCI accuracy varies among participants. Because of this variation, it is difficult to differentiate between the effect of channel selection and the effect of chromatic stimuli when examining the relationship between the imaginary coherence of the MEG data and the P300 performance. Second, we did not collect simultaneous EEG data, and did not evaluate online and offline performance during the P300 tasks. These activities were omitted because a real-time EEG system was not available, and also because the positions of the EEG electrodes available in the simultaneous EEG–MEG recording system are different than in the conventional P300-BCI system. However, it is important to note that a positive relationship was observed between P300 performance and coherence of the EEG data, which was revealed in the comparison between the GB flickering condition and the WG flickering condition.

In our previous research, we found that chromatic visual stimuli improved accuracy in P300-BCI operations (8); in this previous study, the EEG waveform was used to classify the target stimuli. Furthermore, in our previous EEG–fMRI study, we found that peak EEG amplitudes were larger under the GB condition than under the WG condition at the parietal and occipital electrodes (11). These results suggest the ease with which changes can be detected under the GB flicker condition, which is consistent with Polich’s observation that the P300 amplitude is smaller in response to more difficult than to easier tasks (43). Thus, the GB visual stimuli may have allowed participants to detect changes more readily. Furthermore, inter-hemispheric neuronal coherence was improved when the object was recognized (19). We suggest that our chromatic stimuli preferentially elicited right-dominant activation in cooperation with the coherent activities of the bilateral occipital and parietal areas. Taken together, these data support the conclusion that coherent activity in the bilateral parieto-occipital cortices may play a significant role in effectively driving P300-BCI.

## Conflict of Interest Statement

The authors declare that the research was conducted in the absence of any commercial or financial relationships that could be construed as a potential conflict of interest.
